# Correlates of motivation to prevent weight gain: a cross sectional survey

**DOI:** 10.1186/1479-5868-2-1

**Published:** 2005-03-16

**Authors:** Birgitte Wammes, Stef Kremers, Boudewijn Breedveld, Johannes Brug

**Affiliations:** 1 Department of Public Health, Erasmus University Medical Center, Rotterdam The Netherlands; 2 Department of Health Education and Health Promotion, University Maastricht, The Netherlands; 3 Netherlands Nutrition Centre Foundation, The Hague, The Netherlands

**Keywords:** motivation, prevention, weight gain, predictors, stages of change

## Abstract

**Background:**

This study is an application of the theory of planned behaviour (TPB) with additional variables to predict the motivations to prevent weight gain. In addition, variations in measures across individuals classified into Precaution Adoption Process stages (PAPM-stages) of behaviour change were investigated.

**Methods:**

A cross-sectional survey among 979 non-obese Dutch adults aged 25–35 years was conducted. Multiple binary logistic regression analysis was conducted to assess the associations of Body Mass Index (BMI), demographic factors and psychosocial variables from the TPB with the intention to prevent weight gain. Differences in BMI, demographic and psychosocial factors between PAPM-stages were explored using one-way analysis of variance and chi-square tests.

**Results:**

Eighty-five percent of respondents intended to prevent weight gain. Age, attitudes and risk perceptions related to weight gain were the strongest correlates of intention (age: OR = 1.12, 95%CI: 1.04–1.20; attitude OR = 7.91, 95%CI: 5.33–11.74; risk perception OR = 1.24, 95%CI: 1.11–1.38). Significant differences were detected between the PAPM-stages in almost all variables. Notably, perceived behavioural control was lowest among people who had decided to prevent weight gain.

**Conclusion:**

Messages to influence attitudes towards the prevention of weight gain and risk perception may affect people who are not yet motivated to prevent weight gain. Interventions increasing people's perceived behavioural control in overcoming barriers to prevent weight gain may help people to act on their intentions.

## Introduction

Prevalence of overweight and obesity in Western societies has increased rapidly [1]. This is a threat for public health by its link to chronic illness and disabilities and impaired quality of life [2-4]. Overweight is a result of a long-term positive imbalance between energy intake and energy expenditure produced by a relative excess in energy input (diet) and/or a deficit in energy output (physical activity) [5].

In The Netherlands, the prevalence of obesity has approximately doubled over the past 20 years [1]. The prevalence of overweight and obesity is about 45% for men and 35% for women, while 11% of the men and 12% of the women are obese [5,6]. To stop the increase in the prevalence of overweight and obesity, effective strategies for the management and prevention of overweight and obesity need to be developed. Current strategies aimed at treating obesity are not very successful in the long-term [7,8]. The behavioural changes necessary to achieve significant weight loss and maintain a healthy weight are difficult for most people. Therefore, intervention programs aimed to prevent weight gain by encouraging relatively small changes in physical activity and eating habits among people who are not yet overweight may be more promising [6,9,10].

Young adults are of particular interest for prevention of overweight. There is evidence that weight gain is most likely to occur among males and females between the ages of 25 and 34 [6,11,12]. It has also been suggested that the onset of weight gain may have a situational basis, with important events in the life cycle such as entering the workforce, marrying and/ or having children triggering energy imbalance [13]. Prevention of (further) weight gain is also important in young adults who are already overweight.

To support relatively small behavioural changes to reverse the gradual increments in weight with age, it is important to identify correlates of people's motivation to prevent weight gain. Such correlates may be intermediate goals in weight gain prevention programs [14]. The Theory of Planned Behaviour [15] is one of the most widely-applied models to explain health behaviours [16,17]. According to this theory, behaviour in general is determined primarily by behavioural intention and postulates that this intention is determined by three conceptually independent constructs: attitude, subjective norms and perceived behavioural control. Attitudes are the overall evaluations of the behaviour by the individual; subjective norms consist of a person's beliefs about whether significant others think he or she should engage in the behaviour; perceived behavioural control is the individual's perception of the extent to which performance of the behaviour is under voluntary control.

The present study aims to identify potential correlates of motivation to prevent weight gain in a population of young adults in preparation of the development of nation-wide mass media campaigns to prevent weight gain, from the Netherlands Nutrition Centre. Therefore, the present study is a first step to identify potential mediators that should be addressed in such campaigns [18,19]. Primarily, we investigated associations with the constructs from the Theory of Planned Behaviour (TPB) to identify potential correlates of intentions to prevent weight gain in a population of young adults. Additionally, we included constructs that have been identified as a possible extension of the TPB that may be relevant to explain weight maintenance behaviours: descriptive norms (modeling) and social support related to prevention of weight gain, overweight-related risk perceptions, and perceptions of personal weight status. A study by de Vries et al (1995) indicated that there are at least two other categories of social influences besides subjective norms that may determine health behaviour: descriptive norms, i.e. the observed behaviour of others in the direct social environment, and social support, i.e. the direct support people experience for preventive behaviour [20]. Risk perceptions were included as a separate potential determinant in the present study since important alternative health behaviour models such as the Health Belief Model [21] and the Protection Motivation Theory [22] emphasise risk perceptions as a determinant of prevention motivation. Further, weight perception, i.e. self-rated weight status, was included as an additional variable, since there is evidence that people underestimate their weight, which may reduce their perceived need to change and thus their prevention intentions [23-25].

Nowadays, behaviour change is generally conceptualised to occur in subsequent stages or phases [26]. Such stage models like the Transtheoretical model (TTM)[27] and the Precaution Adoption Process Model (PAPM) [28,29] suggest that people in different stages behave in qualitatively different ways and that the content of interventions to encourage people to move toward action should be stage-specific. Transition between stages can be viewed as barriers that must be overcome before action is taken. These stage-models generally distinguish between people who are unaware of or unengaged by a health issue (stage 1), 'engaged and deciding what to do' (stage 2), 'planning to act but not yet acting' (stage 3), 'acting' (stage 4) and 'maintaining' (stage 5). Further, PAPM explicitly distinguished a separate aware but not acting stage, i.e. a step out of the sequence toward action. There is now preliminary evidence that PAPM is applicable to investigate complex behaviour change, like diet and physical activity, and that people in different PAPM stages for these behaviours differ in psychosocial variables like attitudes and perceived behavioural control [30]. Therefore we also investigated how a young adult population was distributed over the different stages related to prevention of weight gain and if people in different PAPM-stages differ in demographic and psychosocial characteristics.

## Methods

### Participants and recruitment

Data for the present study were derived from a cross-sectional survey conducted in November and December 2002. Data collection was performed using telephone questionnaires to obtain representative data of the non-obese Dutch population aged 25–35 years. Telephone numbers were selected at random by means of random digit dialling and stratified to different regions to ensure an equal distribution over the Netherlands.

The participation rate among the population was 71.4% (6587 of the 23053 participants who were called refused to participate to the telephone interview). Those who did not meet the age and BMI inclusion criteria (n = 15449) were excluded. Only respondents aged between 25 and 35 years and with a BMI between 20 and 30 kg/m^2 ^could participate in the study. BMI was calculated from self-reported weight and height. Pregnant women (n = 38) were also excluded from the analysis, since weight gain can and should be expected during pregnancy. This resulted in a study population of 979 respondents of which 56.7% were female; 10.2 % was of non-Dutch origin; 57.5 % had intermediate or less than intermediate vocational training. The mean age was 30.0 years (SD = 3.0) and mean BMI was 23.7 (SD = 2.6), with a 30.1 % prevalence of overweight (BMI ≥ 25).

### Measures

Administration of the telephone questionnaire took approximately 15 minutes. The questionnaire included items of demographics, psychosocial factors related to prevention of weight gain based on the TPB-model, descriptive norm, social support, risk perception and weight perception. Furthermore, the questionnaire obtained measures based on the Precaution Adoption Process Model.

At the start of the interview, the interviewer introduced the topic by explaining that questions would be asked on prevention of weight gain. It was further explained that prevention of weight gain should be understood as 'watching one's weight' by implementing small changes in lifestyle, such as eating habits and physical activity (e.g. reducing the amount of calories of food in meals and snacks and activities to increase amount of physical activity), not only with the purpose to lose weight but particularly in order to avoid gaining weight.

All items to assess TPB and other psychosocial constructs were measured on bipolar five-point scales. The interviewer read out the answering categories. For constructs that were assessed with multiple items, the mean item score was calculated after sufficient internal consistency was established. Cronbach's alphas and inter item correlations were calculated to analyse the internal consistence between the items related to behavioural determinants. Alpha > 0.5 were regarded as acceptable for the present exploratory study [31].

#### TPB constructs

Attitude was assessed directly with three items asking how bad or good; unimportant or important; and unpleasant or pleasant they regarded prevention of weight gain (-2 = very bad; unimportant; unpleasant; 2 = very good; important; pleasant) (α = 0.53). Subjective norm was assessed with one item by asking if respondents thought that 'important others' (e.g. spouse, family, friends, colleagues) wanted them to prevent weight gain (-2 = no, certainly not; 2 = yes, certainly). Perceived behavioural control was assessed with two items asking how difficult or easy respondents thought it is to prevent weight gain is (-2 = very difficult; 2 = very easy) and how certain they were that they can prevent weight gain (-2 = no, certainly not; 2 = yes, certainly) (α = 0.53). Behavioural intention was measured with one item asking the respondent whether they intended to prevent weight gain (-2 = no, certainly not; 2 = yes, certainly).

#### Other psychosocial constructs

Descriptive norm (modeling) was measured by asking respondents to assess how many people they perceived in their direct social environment to actively try to prevent weight gain (-2 = very few people; 2 = almost all people). Social support was measured by asking respondents how much support they received from 'important others' to prevent weight gain (-2 = very little support; 2 = very much support). Risk perception of weight gain was calculated by multiplication of two items, one in which respondents were asked if they believed that they were at less or more risk for weight gain compared to others of same sex, age and height (1 = much less risk; 5 = much more risk) and one item asking how serious they perceived weight gain to be on a three point scale (1 = not serious; 2 = serious; 3 = very serious). This resulted in a risk perception scale of 1–15. Finally, weight perception was assessed by two items, one which asked whether respondents rated their body weight as low or high (-2 = far too light; 2 = far too high) and one in which respondents compared their weight to that of other people of the same sex and age (-2 = much lower weight; 2 = much higher weight) (α = 0.65).

#### PAPM stages of weight gain prevention behaviour

Following Weinstein's method [28], a staging algorithm was used to assign respondents into different PAPM stages (See Table 1).

**Table 1:** PAPM staging-algorithm applied to weight gain prevention

**Respondents in stage:**	**Agreed with statement:**
1. Unengaged	I have never thought about actively trying to prevent weight gain
2. Undecided to act	I have thought about actively trying to prevent weight gain but I do not know (yet) whether I will do so
3. Decided not to act	I have decided not to actively try to prevent weight gain
4. Decided to act	I have decided to actively try to prevent weight gain but I am currently not doing so (yet)
5. Action and maintenance	I do already actively try to prevent weight gain

### Analyses Methods

Multiple logistic regression analysis was conducted in a 3-step process to assess the association of demographic characteristics (age, gender, education, ethnicity and BMI; step 1), TPB constructs (attitude, social norm and perceived behaviour control; step 2) and other psychosocial constructs (modeling, social support, risk perception and weight perception; step 3) with the intention to prevent weight gain. We used logistic regression analyses since we dichotomised the dependent variable 'intention to prevent weight gain' (negative or no intention = 0; positive intention = 1), because of a skewed distribution. Behavioural intention was dichotomised by categorising respondents who answered the intention-question with option 1 (yes, certainly) or 2 (yes, probably) as having a positive intention and those who answered with option -2 (certainly not), -1 (probably not) or 0 (not sure) as having a negative intention. One-way analysis of variance with Scheffe's multiple-comparison test (alpha = .05) and Chi Square (alpha = .05) was used to test for significant differences in demographic and psychosocial factors between the stages of change. All analyses were performed using the SPSS 11.0 statistical program for Windows.

## Results

Eighty-five percent of respondents had a positive intention to prevent weight gain. On average, respondents had positive scores on attitude and perceived behavioural control, negative scores on subjective norm and social support, and neutral scores on descriptive norm, and weight perception (See Table 2).

**Table 2:** Number of items on determinant constructs related to prevention of weight gain, the internal consistency between the items and the mean scores on the constructs, n = 979

Determinant (Range)	Number of items	Internal consistency (α)	Mean score (SD)
*TPB construct:*			
Intention (-2,2)	1		1.36 (1.18)
Attitude (-2,2)	3	0.53	0.59 (0.65)
Subjective norm (-2,2)	1		-0.67 (1.47)
Perceive behavioural control (-2,2)	2	0.53	0.88 (0.92)
*Other psychosocial constructs:*			
Descriptive norm (-2,2)	1		0.20 (1.19)
Social support (-2,2)	1		-1.05 (1.03)
Risk perception (1,15)	2		5.92 (3.69)
Weight perception (-2,2)	2	0.65	0.12 (0.72)

### Predictors of intention to prevent weight gain

With the first step, the logistic regression model explained 11.3% of the variance in intention to prevent weight gain (see Table 3). In this model the variables BMI, sex and age were significant predictors of the intention to prevent weight gain. With the second step, the model was extended with the TPB constructs and explained 45.5% of the variance. Additional to BMI, sex and age, were also attitude and subjective norms significant predictors of the intention to prevent weight gain. With the third step, the regression model was extended with the variables modelling, social support, risk perception and weight perception and explained 49.9% of the variance in intention to prevent weight gain. The results of the likelihood ratio test show that the inclusion of the variables at step 3 significantly increased the explained variance compared with the previous models. In the final model only age, attitude and risk perception were significant predictors of the intention to prevent weight gain (Table 3): respondents were more likely to have a positive intention to prevent weight gain when they were older, had a more positive attitude to prevent weight gain and had higher overweight-related risk perceptions.

**Table 3:** Correlates of intention to avoid weight gain based on stepwise logistics regression analysis (n = 979): odds ratios (OR), 95% confidence intervals (95%CI) and explained variance (Nagelkerke R^2^).

**Step 1 (R^2 ^= 0.113)**		**Step 2 (R^2 ^= 0.455)**		**Step 3 (R^2 ^= 0.494)**	
**Predictor:**	**OR (95%CI)**	**Predictor:**	**OR (95%CI)**	**Predictor:**	**OR (95%CI)**

BMI (20–30 kg/m^2^)	1.30 (1.20–1.42)	BMI	1.22 (1.11–1.35)	BMI	1.04 (0.91–1.19)
Sex (0 = man; 1 = woman)	2.34 (1.61–3.39)	Sex	1.81 (1.13–2.89)	Sex	1.00 (0.58–1.71)
Age (25–35y)	1.07 (1.01–1.13)	Age	1.09 (1.01–1.17)	Age	1.12 (1.04–1.20)
Education (1 = low; 2 = high)	1.29 (0.92–1.81)	Education	1.26 (0.89–1.78)	Education	1.19 (0.84–1.70)
Ethnicity (1 = Non Dutch origin; 2 = Dutch origin)	1.04 (0.58–1.89)	Ethnicity	1.22 (0.60–2.50)	Ethnicity	1.23 (0.58–2.59)
		Attitude	10.10 (6.85–14.89)	Attitude	7.91 (5.33–11.74)
		Subjective norm	1.24 (1.04–1.47)	Subjective norm	1.17 (0.97–1.40)
		Perceived behavioural control	1.00 (0.78–1.27)	Perceived behavioural control	1.12 (0.88–1.44)
				Descriptive norm	1.19 (0.99–1.45)
				Social support	1.10 (0.82–1.47)
				Risk perception	1.24 (1.11–1.38)
				Weight perception	1.36 (0.81–2.30)

### Differences in factors across PAPM stages of change

Table 4 shows that a majority of the respondents reported that they already act to prevent weight gain. Significant differences were detected between the PAPM stages in almost all socio-demographic and psychosocial variables with the exception of educational level and descriptive norms. Respondents who were in the decided-to-act and the action stage were on average older, more likely to be female and had a higher BMI compared with respondents in earlier stages. Furthermore, the respondents who were in the decided-to-act and action stage had a more positive attitude towards prevention of weight gain, reported a higher subjective norm, perceived more social support to prevent weight gain, had a higher overweight-related risk perception and evaluated their weight as significantly higher compared to those in the earlier stages. Finally, the results showed that the respondents who were in the decided-to-act stage reported a lower perceived behavioural control towards prevention of weight gain than respondents in most other stages.

**Table 4:** Significant differences between PAPM stages on socio-demographic and psychosocial correlates (n = 979)

	**UE **N = 109 (11.1%)	**UD **N = 64 (5.1%)	**DN **N = 80 (8.2%)	**DA **N = 85 (8.7%)	**A **N = 641 (65.5%)	Significant differences*
***Demographic variables:***						
Age	30.00	30.28	30.35	31.28	30.98	A>UE
Sex (% women)	30.3	29.7	37.5	54.1	64.1	DA, A>UE, UD A>DN
Education (% higher education)	40.4	60.9	45.0	47.1	53.0	-
BMI	22.33	23.12	23.02	24.79	23.97	DA> UE, UD, DN A> UE, DN
***TPB constructs:***						
Attitude	-0.095	0.35	-0.079	0.42	0.83	A> UE, UD, DN, DA UD, DA >DN, UE
Subjective norm	-1.06	-0.88	-1.14	-0.28	-0.57	A, DA > UE, DN
Perceived behavioural control	0.82	0.87	0.74	0.38	0.96	UE, UD, A > DA
***Other psychosocial constructs:***						
Descriptive norm	-0.028	-0.078	-0.063	0.035	0.31	-
Social support	-1.49	-1.42	-1.54	-1.02	-0.88	A> UE, UD, DN DA> UE, DN
Risk perception	2.96	3.97	3.89	6.51	6.79	DA, A > UE, UD, DN
Weight perception	-0.46	-0.094	-0.22	0.46	0.23	DA, A > UE, UD, DN UD>UE

*Statistical significant at *P *< 0.05

*Note: *UE = unengaged; UD = undecided to act; DN = decided not to act; DA = decided to act; A = action

## Discussion

The present study shows that a majority of Dutch non-obese adults aged between 25 and 35 years had a positive intention to prevent weight gain. Age, attitudes and risk perceptions related to weight-gain were the strongest predictors of this intention. Further, a majority of the respondents reported that they were already acting to prevent weight gain and respondents in the higher stages of change reported more positive psychosocial factors related to weight-gain prevention than respondents in earlier stages of change. However, respondents who had decided to act to prevent weight gain reported low perceived behavioural control, suggesting that low control was a barrier towards action.

The study indicates that in order to motivate the minority with non-positive intentions to prevent weight gain, interventions should focus on attitude change and communication about the risks of weight gain and overweight. In order to help motivated people in the study population to act on their intentions, it may be important to increase perceived behavioural control. These results are in line with earlier studies in differences on psychosocial factors between stages of change for smoking cessation [32], fat reduction [33], and increasing fruit and vegetable consumption [34], that also concluded that attitudinal information and risk information should be provided in earlier stages of change to increase motivation, while self-efficacy or control information can assist people to move to the action stage. The importance of perceived behavioural control is also highlighted in studies on weight loss [35,36].

Our results further show that people who have decided to act have higher BMIs than people in earlier stages of change, which shows that people who have more reason to act may also be more ready for action. However, this result is not supportive to the 'prevention of overweight' goal, since it indicates that people become more inclined to prevention of weight gain when they already experience some overweight. The fact that women were more likely to be in later PAPM stages reflects results from earlier studies that show that women are more weight conscious [37,38]. The result from a secondary *t*-test analyses showed that women in the present study were significantly more weight concerned compared to men, which may also explain why the association of sex with the intention to prevent weight gain was non significant after adjustment for weight perception.

The present study shows that a large majority of respondents regarded themselves as acting to prevent weight gain. However, this does not tell us if they have been successful in their self-perceived actions and if their actions would prevent weight gain in the future. Studies conducted in the Netherlands have shown that the prevalence of overweight is still increasing [1]. Taking this trend into account, the results of this study suggest that it is likely that respondents overestimated their actions to prevent weight gain or that they applied less effective strategies to balance their energy intake and expenditure. The present study further shows that about a quarter of respondents were still in the earlier stages of behaviour change. They may have perceived less need to prevent weight gain, since most of the respondents do not yet have a weight problem. This illustrates the challenge of prevention of weight gain: we need to encourage the non-overweight population to be attentive to and to act to prevent gradual changes in weight.

For interpreting the results of this study, several limitations should be acknowledged. Firstly, the present study relied on self-reports, which may be subject to social desirability bias. No objective assessments of energy balance related behaviours were included in our measurements. Earlier studies show that errors in self-reporting increases directly with the magnitude of overweight and with age after the age of 45 years [39,40]. Since we studied a non-obese younger population group, self report-bias may have been limited but cannot be ruled out.

Our measures of TPB and PAPM constructs were in accordance with those used in earlier studies [15,26], which were further pilot tested in a small sub-sample of the study population and submitted for review to an expert panel. For the present study, we framed all questions as related to 'watching your weight in order to prevent weight gain'. This formulation may not have been specific enough to embrace the complexity of energy balance related behaviour. Furthermore, some measures were based on few or single item assessments, which are more likely to have limited reliability. For future intervention development it will be more informative to investigate indirect measures of behavioural determinants, e.g. to identify what sort of specific beliefs may underlie people's weight gain prevention attitudes and control beliefs. For example, other studies indicate that people believe that low fat diets (an illustration of a specific weight control behaviour) are more expensive, more difficult to prepare and that eating less fat is difficult in general or in certain specific situations such as in the weekends and holidays [41-43]. In order to tailor education interventions, qualitative methods such as interviews or focus groups are needed to gain more knowledge about existing underlying salient beliefs of the target population [18]. This to determine which beliefs have to be changed by introducing new salient beliefs or by reinforcing existing beliefs [44].

Furthermore, we applied a cross-sectional research design and therefore were only able to study associations, and not causation [45]. Despite this limitation, results of meta-analyses revealed that TPB accounts for about 41% of the variance in intentions and 34% of the variance in behaviour across a variety of health-related behaviours [16]. These findings may establish to the credibility of behavioural intentions as the main predictor for weight maintenance-actions. Further, we also have to acknowledge limitations of the analyses since so many (85%) of the whole sample was found as having a positive intention. Because of this high percentage, we conducted additional analyses with more conservative data by classifying only those answering 'yes, certainly' as having a positive intention. The results of these analyses revealed that in the final step of the regression model, additional to the variables 'attitude' and 'risk perception' the variables 'sex', 'social support' and 'weight perception' became significant predictors of the intention to prevent weight gain. This means that respondents were also more likely to have a positive intention to prevent weight gain when they were female, be supported more by others and when they rated their weight as higher. For intervention development, these variables may be taken into consideration in order to motivate people who already have positive intentions to prevent weight gain, but who are still undecided on whether they will take action in the near future. Finally, the present study included only relatively few respondents in the earlier stages of behavioural change, and thus statistical power to detect differences between these stages was low. We therefore were not able to show with the present study that the PAPM was more useful than the TTM in order to investigate prevention of weight gain. Nevertheless, the PAPM might give additional information compared to TTM assessments when it is applied to more specific weight related behaviours.

Our study population was a random sample of non-obese 'young' adults in the age group of 25–35 year. The mean BMI of our study population was indeed identical to that of the Dutch population aged 25–35 at large, although women were slightly over-represented and the respondents in this study were somewhat higher educated compared with the source population [46,47]. However, this is unlikely to have serious consequences for the generalisability of the findings, as results of the present study show that sex and educational level were not significant correlates of the motivation to prevent weight gain.

Maybe the most striking result of this study is the high proportion of positive intentions to prevent weight gain and the high prevalence of respondents who reported to act in order to prevent weight gain. In this age group, weight gain is highly probable. The present study indicates that this occurs in spite of high motivation and self-perceived actions to avoid gaining weight. Andajani-Surjahjo and colleagues recently reported a lack of motivation related to physical activity and healthy eating for weight maintenance among women in the US [48]. This may illustrate the issue that people are motivated to avoid weight gain, but less motivated to engage in more specific actions that help to accomplish weight management. For a further understanding of the correlates of prevention of weight gain we need to investigate motivational factors (e.g. attitudes; subjective norms etc) related to specific weight-management actions such as 'avoiding calories', limiting the amount of food, and participation in regular physical activities [49]. Furthermore, we should not restrict our investigations to motivational factors. In the last decade, a number of reports have been published arguing that our present society fosters an 'obesogenic environment', i.e. a physical and social environment that promotes over-eating and discourages physical activity, and that such an environment may provide too high a barrier to avoid becoming overweight, despite high prevention motivation [50]. Further research is needed to investigate which energy balance related behaviours can help people deal better with the 'obesogenic environment' and may enable them to effectively act on their positive weight-gain prevention intentions.

## Competing Interests

The author(s) declare that they have no competing interests.

## Authors' Contributions

BW conceptualised the study, performed the statistical analyses, and drafted the manuscript. SK and BB both participated in the conceptualisation of the study, interpretation of the results and helped to draft the manuscript. JB conceived the study and assisted in the interpretation of the results and drafting the manuscript. All authors read and approved the final manuscript.
